# Multiple Serotypes of Bluetongue Virus in Sheep and Cattle, Israel

**DOI:** 10.3201/eid1612.100239

**Published:** 2010-12

**Authors:** Jacob Brenner, Chris Oura, Itai Asis, Sushila Maan, Dani Elad, Narender Maan, Orly Friedgut, Kyriaki Nomikou, Ditza Rotenberg, Valizar Bumbarov, Peter Mertens, Hagai Yadin, Carrie Batten

**Affiliations:** Author affiliations: Kimron Veterinary Institute, Bet Dagan, Israel (J. Brenner, D. Elad, O. Friedgut, D. Rotenberg, V. Bumbarov, H. Yadin);; Institute for Animal Health, Pirbright, UK (C. Oura, S. Maan, N. Maan, K. Nomikou, P. Mertens, C. Batten);; Hachacklait, Caesarea, Israel (I. Asis)

**Keywords:** Bluetongue virus, diagnosis, real-time PCR, Israel, multiple serotypes, sheep, cattle, viruses, letter

**To the Editor:** In September 2008, the Israeli Veterinary Field Services were notified of uncharacteristic disease on a dairy farm near the border with Lebanon in Rosh Ha Nikra, (Figure A1). In November, blood samples were obtained from 5 cows, 4 of which were recovering from signs of infection with bluetongue virus (BTV). Virus isolation was conducted at the Kimron Veterinary Institute, Bet Dagan, Israel. One isolate (ISR2008/03) was sent to the World Animal Health Organisation Bluetongue Reference Laboratory at the Institute for Animal Health (IAH), Pirbright, UK, for further characterization. BTV-16 was identified by using serotype-specific real-time reverse transcription–PCR (RT-PCR) for genome segment 2 (Seg-2). BTV-16 has been detected in Israel and is considered endemic, along with BTV serotypes 2, 4, 6, and 10 (*1*).

Ten additional blood samples and 1 spleen sample subsequently obtained from affected cattle on the farm were sent to IAH. All samples were tested for BTV by serogroup-specific real-time RT-PCR specific for Seg-1. Six samples (including 1 from the spleen) were positive for BTV. Serotype-specific real-time RT-PCR showed that 2 blood samples contained BTV-16 and 1 blood sample contained BTV-4 and BTV-16. The amount of BTV RNA in the remaining 3 RT-PCR–positive samples was low, and attempts to identify serotype were unsuccessful. Virus from the spleen was isolated in an insect cell line (KC cells from *Culicoides sonorensis* midge embryos, CRL 1660; American Type Culture Collection, Manassas, VA, USA), and the virus was serotyped as BTV-8 by RT-PCR.

BTV-4 was isolated from bovine blood obtained in October 2008 from a farm in Zde Eliahu, 100 km east of Rosh Ha Nikra. However, this animal was co-infected with BTV-24, which has been found at numerous sites in Israel (Figure A1). BTV-24 was isolated at IAH from samples obtained from sheep and cattle showing clinical signs of disease. BTV-4, BTV-16, and BTV-24 all reemerged in Israel during 2009, the mortality rate was up to 80% on 1 sheep farm infected with BTV-24 (*2*). An outbreak in Hatzafon in November 2009 was confirmed as BTV-5 by serotype-specific real-time RT-PCR.

To determine origins of BTV strains causing these outbreaks, we sequenced Seg-2 of the BTV-4 (Zde Eliahu) and BTV-8 and BTV-16 (ISR2008/02, ISR2008/13, and ISR2008/03) isolates from Israel. BTV-16 ISR2008/03 had >99% nt sequence identity (2,935 bp) with BTV-16 (GRE1999/13) isolated in Greece in 1999 but was distinct from BTV-16 (OMN2009/02) recently isolated in Oman. BTV-8 isolate ISR2008/13 had >99% nt sequence identity (2,939 bp) with the northern European strain of BTV-8 (NET2006/04). This finding indicates that the BTV-8 isolate from Israel (ISR2008/13) belongs to the same lineage as BTV-8 from northern Europe (NET2006/04) and may have been introduced into Israel during importation of BTV-8–positive animals from northern Europe.

BTV-4 isolate ISR2008/02 had >99% nt sequence identity (2,926 bp) with BTV-4 (DQ191279) isolated in Israel in 2001, which suggests that this serotype has either continued to circulate or has reemerged. BTV-24 (ISR2008/05) belongs to a western topotype. However, few nucleotide sequences are available for comparison of BTV-24 Seg-2 regions. BTV-5 has not been isolated; therefore, no sequence data are available.

Although BTV-2, BTV-4, BTV-6, BTV-10, and BTV-16 are considered endemic to Israel, clinical signs of disease are uncommon. We report clinical signs of infection in cattle in Israel caused by BTV-8 and BTV-24. We also report active circulation of 5 BTV serotypes (BTV-4, BVT-5, BTV-8, BTV-16, and BTV-24) during 2008–2009. Multiple serotypes were isolated on 3 farms containing sheep that had clinical signs of BT (farm 1: BTV-4 and BTV-24, farm 2: BTV-8 and BTV-24, and farm 3: BTV-4, BTV-8, and BTV-24). BTV-4, BTV-8, and BTV-16 were also isolated from cattle at Rosh Ha Nikra. Identification of multiple cocirculating BTV serotypes increases the likelihood of genome segment reassortment, which could potentially lead to increased virulence. Whole genome sequencing of isolates from these farms is in progress to determine whether any of these isolates are reassortants, as has been observed in Italy (*3*)

Our study indicates that BTV-8 strains from Israel and northern Europe (*4**–**6*) are closely related and share a recent common origin. The strain from Israel may represent an extension of the outbreak in Europe. Use of inactivated virus vaccines has dramatically decreased the number of cases caused by virulent BTV-8 in Europe (*7*), which suggests that a similar campaign might be effective in Israel. However, the BTV-24 strain from Israel appears to be highly virulent in cattle and sheep, and absence of a live or inactivated vaccine against this serotype could lead to its reemergence and to severe economic losses. In the absence of an appropriate vaccine and control strategy, the virus could potentially spread to neighboring countries and pose an additional risk to Europe.

## Figures and Tables

**Figure A1:**
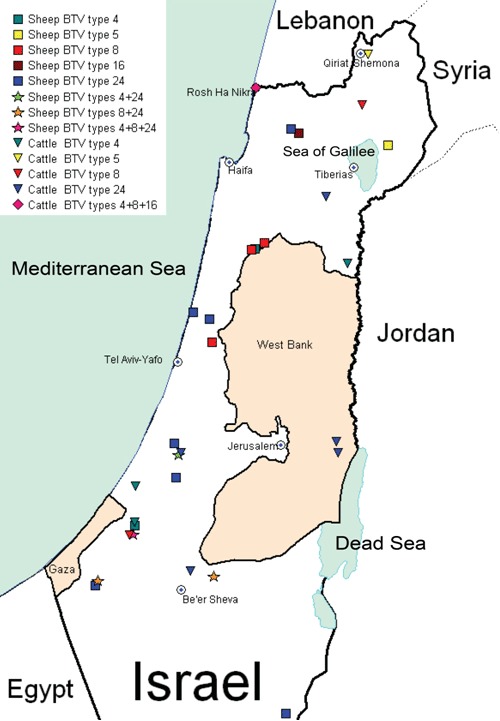
Bluetongue virus (BTV) serotypes identified in Israel, 2008–2009.
